# Exercise-induced Laryngeal Obstruction: Protocol for a Randomized Controlled Treatment Trial

**DOI:** 10.3389/fped.2022.817003

**Published:** 2022-02-07

**Authors:** Hege Clemm, Ola D. Røksund, Tiina Andersen, John-Helge Heimdal, Tom Karlsen, Magnus Hilland, Zoe Fretheim-Kelly, Karl Ove Hufthammer, Astrid Sandnes, Sigrun Hjelle, Maria Vollsæter, Thomas Halvorsen, Bergen ILO-group, Haakon Kvidaland

**Affiliations:** ^1^Department of Clinical Science, University of Bergen, Bergen, Norway; ^2^Department of Pediatric and Adolescent Medicine, Haukeland University Hospital, Bergen, Norway; ^3^Faculty of Health and Social Sciences, Western Norway University of Applied Sciences, Bergen, Norway; ^4^Department of Otolaryngology and Head and Neck Surgery, Haukeland University Hospital, Bergen, Norway; ^5^Department of Physiotherapy, Haukeland University Hospital, Bergen, Norway; ^6^Norwegian Advisory Unit on Home Mechanical Ventilation, Thoracic Department, Haukeland University Hospital, Bergen, Norway; ^7^Department of Surgery, Haukeland University Hospital, Bergen, Norway; ^8^Department of Clinical Medicine, University of Bergen, Bergen, Norway; ^9^Faculty of Veterinary Medicine, Norwegian University of Life Sciences, Oslo, Norway; ^10^Centre for Clinical Research, Haukeland University Hospital, Bergen, Norway; ^11^Department of Internal Medicine, Innlandet Hospital Trust, Gjøvik, Norway; ^12^Department of Sports Medicine, Norwegian School of Sport Sciences, Oslo, Norway

**Keywords:** EILO, VCD, exercise, RCT (randomized controlled trial), protocol, treatment

## Abstract

**Background:**

Exercise-induced laryngeal obstruction (EILO) is a common cause of exertional breathing problems in young individuals, caused by paradoxical inspiratory adduction of laryngeal structures, and diagnosed by continuous visualization of the larynx during high-intensity exercise. Empirical data suggest that EILO consists of different subtypes, possibly requiring different therapeutic approaches. Currently applied treatments do not rest on randomized controlled trials, and international guidelines based on good evidence can therefore not be established. This study aims to provide evidence-based information on treatment schemes commonly applied in patients with EILO.

**Methods and Analysis:**

Consenting patients consecutively diagnosed with EILO at Haukeland University Hospital will be randomized into four non-invasive treatment arms, based on promising reports from non-randomized studies: (A) standardized information and breathing advice only (IBA), (B) IBA plus inspiratory muscle training, (C) IBA plus speech therapy, and (D) IBA plus inspiratory muscle training and speech therapy. Differential effects in predefined EILO subtypes will be addressed. Patients failing the non-invasive approach and otherwise qualifying for surgical treatment by current department policy will be considered for randomization into (E) standard or (F) minimally invasive laser supraglottoplasty or (G) no surgery. Power calculations are based on the main outcomes, laryngeal adduction during peak exercise, rated by a validated scoring system before and after the interventions.

**Ethics and Dissemination:**

The study will assess approaches to EILO treatments that despite widespread use, are insufficiently tested in structured, verifiable, randomized, controlled studies, and is therefore considered ethically sound. The study will provide knowledge listed as a priority in a recent statement issued by the European Respiratory Society, requested by clinicians and researchers engaged in this area, and relevant to 5–7% of young people. Dissemination will occur in peer-reviewed journals, at relevant media platforms and conferences, and by engaging with patient organizations and the healthcare bureaucracy.

## Introduction

Paradoxical closure of the larynx during physical exertion, or exercise-induced laryngeal obstruction (EILO), has now been recognized as a relatively common cause of exertional breathing problems in particularly young and physically active individuals. Studies report prevalence rates of 5–7% in unselected adolescent populations (1, 2), and even higher in groups where exercise is particularly important (3, 4). EILO hampers participation in physical activity, and can in severe cases lead to disabling exercise-related dyspnoea. Diagnostic confusion between EILO and exercise-induced asthma likely leads to overuse of asthma medication and underuse of EILO treatment (5–8).

Laryngeal function during exercise is not fully understood, which hampers our approach to the treatment of EILO. Empirical data suggest that EILO consists of different subtypes that might require different therapeutic approaches. However, despite several studies suggesting positive treatment responses, widely applied therapeutic approaches still do not rest on randomized controlled trials (9–14). Randomized clinical trials (RCT) are pivotal in guiding clinical practice, and valid diagnostic methods and outcome measures are vital for their applicability (15). No RCTs have in fact been performed in this area, and it was therefore challenging to decide where to focus our efforts. We have designed a trial that will answer questions we find imperative and involves treatments used in our own department based on accumulated clinical experience. The overarching aim of this study is to provide evidence-based information on interventions commonly applied to treat EILO.

### EILO Diagnostic Methods and Graded Outcome Measures

**Figure 1:**
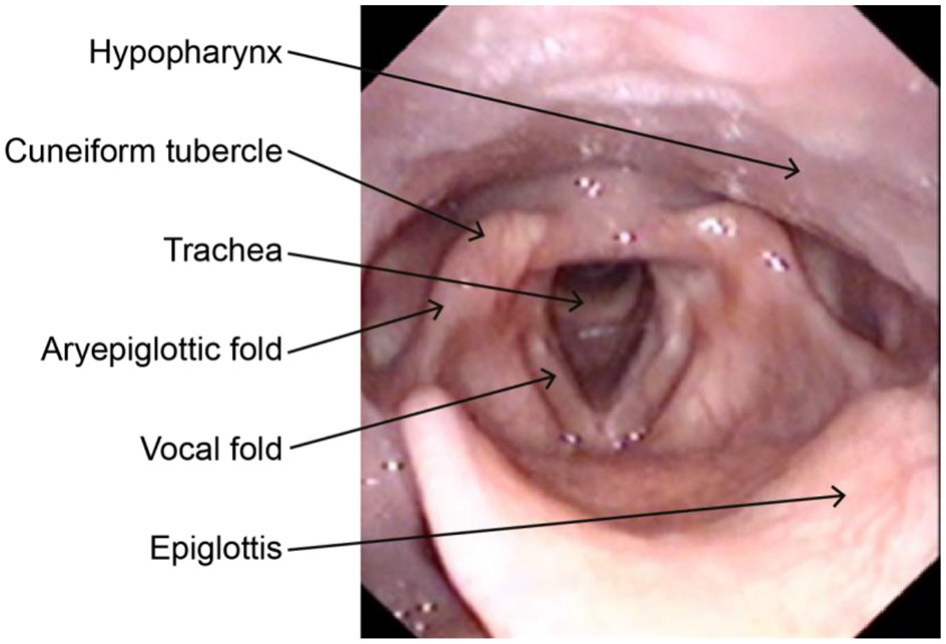
Laryngeal anatomy with relevant landmarks as seen through a laryngoscope.

EILO is diagnosed by laryngoscopy performed continuously from rest to peak exercise, a test labeled continuous laryngoscopy exercise (CLE) test (13, 16, 17). The test provides visualization of the level of the obstruction within the larynx; i.e., glottic, supraglottic (or both), and epiglottic obstruction (Figure 1), and permits timing of the events; i.e., what laryngeal structure that incites the adduction. By definition, EILO is inappropriate inspiratory laryngeal adduction that occurs during ongoing exercise in a larynx that appears normal while at rest (13). The CLE scores assess *the relative* degree of inspiratory adduction of the supraglottic and vocal fold during increasing exercise (18). As CLE scores rest on judgements made by raters, there is inevitably an aspect of subjectivity involved when rating of EILO is performed. Studies addressing the validity of this CLE scoring system have reached somewhat variable conclusions (18–20). It is reasonable to conclude from the literature that experience is a key factor that influences the repeatability of the rater. Although the CLE scoring system has its shortcomings, it is to date the most objective measure of EILO severity, and it is regularly used in everyday clinics worldwide.

Our group recently published results showing that translaryngeal pressure measurements performed throughout the CLE test is a feasible procedure in healthy volunteers. Combined with flow measurements obtained by the mouth, translaryngeal resistance is a promising objective numerical measure that can be used in the evaluation of patients with EILO (21). Objective numerical measures are of particular interest in cases where supraglottoplasty is considered, as surgery is an irreversible procedure involving (as all surgery) aspects of risk. As surgery leaves obvious marks in the larynx, blinding of raters will not be possible in research. Therefore, objective measures of resistance is highly welcomed.

### Treatment of EILO

The European Respiratory Society (ERS), the European Laryngological Society (ELS) and the American College of Chest Physicians (ACCP) joint Task Force Statement on EILO (13, 16) conclude the chapter on therapy by stating that future treatment studies must: “*Acknowledge the complex nature of laryngeal form and function, carefully categorise the patients by the new descriptive taxonomy, at least discriminate between laryngeal obstruction occurring at rest and during exercise, and whether glottic or supraglottic structures are primarily involved. Interventions and study outcomes must be clearly defined, and participants randomized into treatment and control arms.”*

Non-invasive treatments include strategies such as simple breathing advice, speech therapy (22, 23), biofeedback (24, 25), inspiratory muscle training (IMT) (10, 26), and laryngeal control therapy (23, 27). EILO subtypes are often not defined in studies, and the exact treatment methods are often poorly detailed. These issues complicate interpretations and question the generalizability of the findings (28). Our department has clinical experience with most types of treatment, in particular breathing advice, speech therapy, and IMT, as well as surgery offered to highly selected cases of motivated patients with significant supraglottic EILO, using laser supraglottoplasty.

### The Specific Aim of This Study

The specific aim of this study is to test the effect of four different non-invasive treatment approaches in a randomized controlled design in patients consecutively diagnosed with EILO at Haukeland University Hospital. Due to financial reasons, this is a one-center only study. Findings from continuous laryngoscopy performed during maximal treadmill exercise (CLE) will be used to diagnose and subtype EILO, and CLE-scores will be used to assess changes in the larynx at peak exercise before vs. after the interventions. In patients failing the non-invasive treatment approach, and who otherwise qualify for surgical intervention, two surgical approaches will be tested against a “*wait-and-see*” control group, with translaryngeal resistance measured before and after as an added numerical objective outcome measure.

## Methods

### Subjects and Design

**Figure 2:**
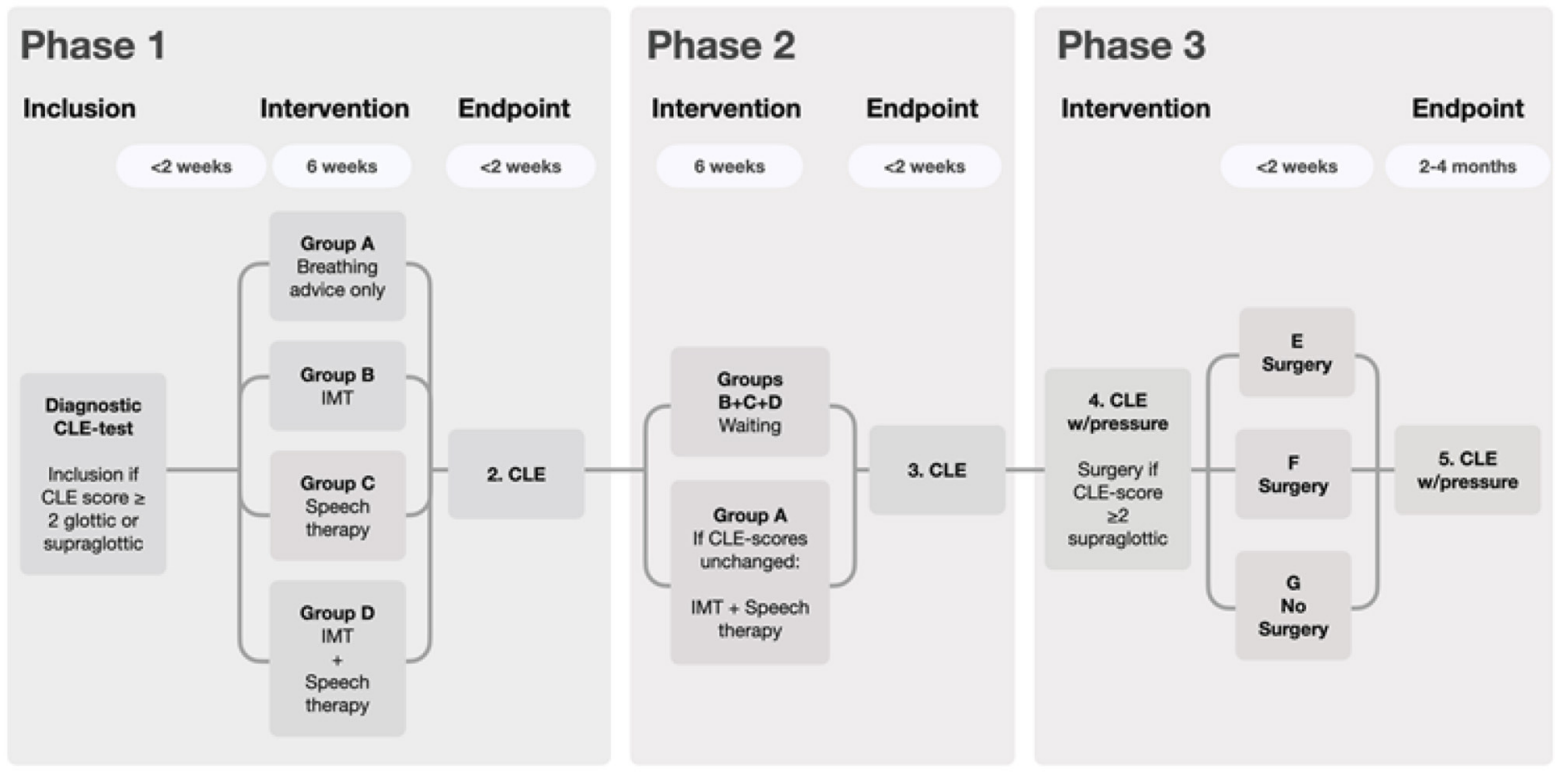
Flow chart depicting the four non-invasive approaches and two different surgical approaches. During Phase 1, breathing advice will be provided to all groups, and constitutes the only measure in group A, serving as reference for the groups B, C, and D.

The study will be conducted as a randomized controlled trial, performed at the EILO clinic at Haukeland University Hospital in Bergen, Norway, a nationwide referral center, annually seeing ~200 new patients. All study participants will be recruited from this EILO clinic. The design is outlined in Figure 2. After the diagnostic CLE test, all eligible patients will receive written information about the study. A nurse, not otherwise involved in the study, will provide oral information and be available for questions. Consenting participants will sign a written consent form before inclusion. For patients under the age of 16, their parents must also sign the form. Patients who decline participation will be offered treatment according to present department strategy; i.e., based on a “best practice” approach and according to current department policy for follow-up. We will need 140 patients in phase 1 and 30 patients in phase 3 (see statistics). However, our clinical experience is that ~10% of EILO patients are referred for surgery. The total number of included patients will then be 350.

The regional ethical committee of Western Norway approved the study (REK 2020-134444), and the study is registered in Clinical trials 2020/134444.

### Inclusion Criteria

- EILO with CLE score at peak exercise graded as ≥2 at glottic *or* supraglottic level **and**
- Respiratory complaints to an extent that the patient feels a need for further treatment and follow-up.

### Exclusion Criteria

- Breathing problems other than EILO, except well-controlled asthma.
- Perceived to be unable to perform repeated maximal cardiopulmonary treadmill exercise tests, or failing to accept the procedures required for repeated successful CLE tests, or unable to perform any of the other examinations required by the protocol.
- Abnormal anatomy at rest in the laryngeal region or the upper airways.
- Age below 12 years.

## The Study Phases

**Phase 1:** This is the main treatment period.

Consenting patients, irrespective of EILO subtype, will randomly be allocated into one of four treatment arms:

A. Basic information and breathing advice with biofeedback (IBA). This is the reference treatment against which the other methods will be measured.
B. IBA plus inspiratory muscle training (IMT).
C. IBA plus speech therapy.
D. IBA plus both inspiratory muscle training and speech therapy.

**Phase 2:** Patients from group A (the reference group) who still fulfill the inclusion criteria will be offered treatment corresponding to group D; i.e., both IMT and speech therapy. For the other groups, Phase 2 will consist of waiting, testing the long-term effects of any effects gained from the interventions performed in Phase 1. Participants with no further complaints and who do not fulfill the criteria for surgery will stop follow-up after Phase 2.

**Phase 3:** Patients whose *supraglottic* CLE score still ≥2 and who otherwise fulfill the criteria for surgical intervention despite non-invasive treatment, will be offered to be randomized to (E) supraglottoplasty—full procedure or (F) supraglottoplasty minimally invasive procedure or (G) no surgery; the latter serving as the reference group for (E) and (F).

### Questionnaires

All patients will complete custom-made questionnaires recording demographic background variables and symptom scores; for details. The questionnaires focus mainly on respiratory symptoms experienced by the patients, treatment they have been exposed to, and diagnoses they have been assigned. Relevant co-morbidities will also be recorded.

## Detailed Descriptions Of The Applied Test Set-Ups

### Pulmonary Function and Exercise Test

Spirometry will be performed with Vyntus and MasterScreen on SentrySuite software (Vyaire Medical, Hoechberg, Germany) according to guidelines (29). The following expiratory and inspiratory volumes and flows will be recorded: Forced expiratory and inspiratory volume capacity (FVC and FIVC), forced expiratory and inspiratory volume in first second (FEV1 and FIV1), forced expiratory and inspiratory flow at 50% of FVC (FEF and FIF50) and at 25–75% of FVC (FEF and FIF25-75). Whenever possible, raw data will be standardized for age, height, and sex and presented as z-scores and percent predicted (30). The inspiratory and expiratory configuration of the flow-volume loops will be recorded and classified. Prior to testing, the equipment will be calibrated according to the manufacturer's guidelines.

### Continuous Laryngoscopy Exercise Test (CLE-Test)

A diagnostic CLE test will be performed as part of the standard workup for all patients received at the EILO clinic. All study participants will run to symptom-limiting distress or exhaustion on a treadmill (Woodway ELG 70, Weil am Rhein, Germany), wearing a head-set and face mask (Hans Rudolph, Inc., Kansas City, MO, USA) to secure a transnasal fiberoptic flexible laryngoscope (Olympus, Tokyo, Japan) in the best possible epipharyngeal position, ensuring a good view of the larynx. The patient is connected to a Vyntus cardiopulmonary exercise (CPX) unit (Vyaire Medical) and a 12-lead ECG (Marquette Medical Systems Inc., Milwaukee, WI, USA). An integrated set-up combines the data from the CPX unit and the laryngoscope, as well as a soundtrack and a video recording of the upper part of the body. The treadmill will be run according to a computerized protocol, incrementing speed, and/or grade every 1 min, aiming to obtain peak oxygen uptake after 6–12 min of exercise. All information will be stored in a computer file for later assessment. The test will be considered successful if the patient reproduces his or her respiratory complaints, or indicate exhaustion, preferably supported by a plateau in oxygen consumption and/or heart rate, and respiratory exchange ratio (RER) exceeding 1.10.

#### The CLE Scoring Procedure

**Figure 3:**
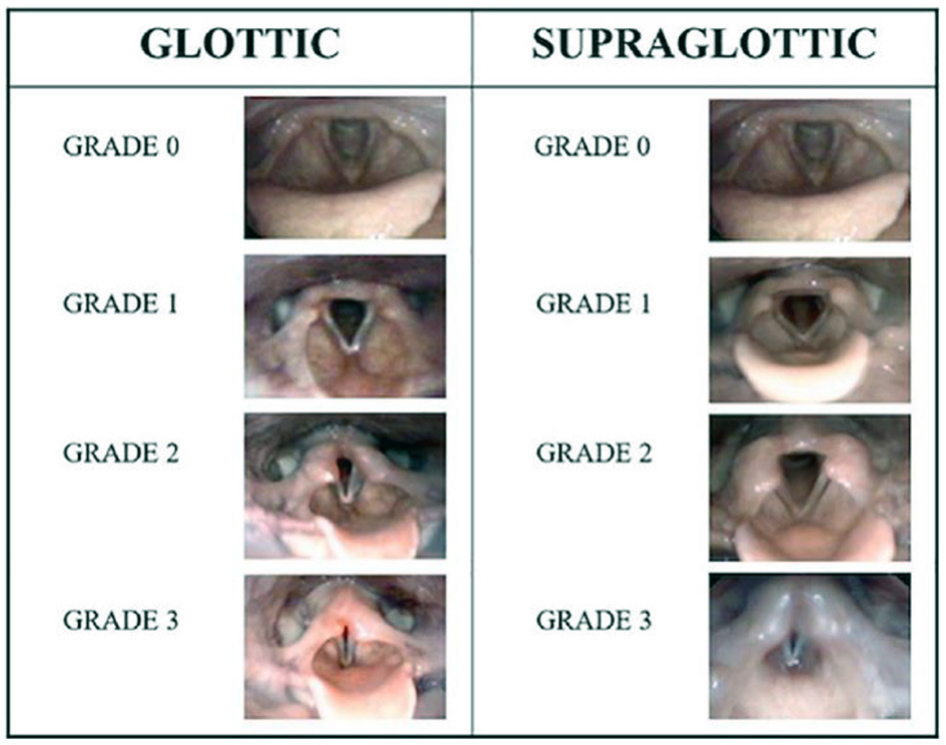
CLE grading system, reproduced with permission from Fretheim Kelly et al., Frontiers in Physiology (31).

Two highly experienced raters will score all CLE tests according to a system that has been published previously (18). EILO will be graded based on the severity of the visual obstruction of the supraglottic and glottic structures during exercise, with scores from 0 (complete patency) to 3 (almost complete closure) at the glottic and supraglottic level at moderate and at maximum intensity (Figure 3) (2, 18, 19). Both raters have assessed CLE recordings from ~3,000 individuals (18). The raters will review the video recordings retrospectively and not in the test situation, with recordings presented to each rater blinded to the identity of the patient and to whether the recordings were obtained before or after the intervention. Cases where scores differ between the two raters will be solved by consensus.

#### Measuring Translaryngeal Resistance

Patients eligible for surgical intervention (Figure 2) will perform CLE tests that include also measurements of translaryngeal resistance, thus providing outcome measures not subject to any form of bias by the raters. The method has been described in a previous communication (21). Briefly, calculation of resistance is based on pressure recordings obtained from two pressure sensors placed above and below the larynx, and the airflow measured at the mouth. The vocal fold and proximal trachea will be anesthetized by lidocaine administered *via* an Olympus Spray tip catheter PW-6C-1 producing a lidocaine mist. Two pressure sensors (Mikro-Cath 825-0101, Milar, Houston, USA) will be introduced through a work channel in the laryngoscope, the first positioned approximately corresponding to the fifth tracheal ring and the second at the tip of the epiglottis. Both sensors are connected to a data acquisition box (Powerbox 8/35, AdiInstruments, Oxford, UK), collecting the data at 40 Hz, stored on a MacBook laptop using LabChart 8.0 software. Maximum inspiratory translaryngeal resistance will be determined at exhaustion; i.e., using ~10 breaths during the last 10 s of the CLE test, using this equation: [RL = PT – PE/AF] where RL is Laryngeal resistance (cm H_2_O/Ls-1), PT is Tracheal pressure reading (cm H_2_O), PE is Epiglottic pressure reading (cm H_2_O), AF is Airflow in Liters per second.

## The Treatment Interventions

### Information and Breathing Advice With Bio-Feedback (IBA)

Information and breathing advice (IBA) involving also an element of biofeedback will be provided to all groups. IBA has been provided in a relatively similar way to all patients diagnosed with EILO at our clinic during the past ~10 years, irrespective of provision of any other forms of treatment. This will not be altered in this study. Thus, a standardized version of our “IBA with biofeedback” treatment will serve as an “active comparator” treatment arm. The maximum time allowed for IBA and biofeedback in this study will be 30 min. The teaching will be provided by the attending physician and the test leader together. There will be a checklist for each patient, ensuring that all elements are covered, and provided similarly to all patients.

#### Before Starting the CLE-Test

After the laryngoscope has been secured in the correct position (see above, CLE-test), the patient will be shown his/her larynx on the monitor. The test leader points out important anatomical structures and landmarks, like the base of the tongue, the epiglottis, the cuneiform tubercle, the aryepiglottic fold, the true and false vocal cords and the tracheal inlet. The patient will then be asked to perform various breathing manoeuvers while observing his/her larynx. First: Breathe normally, then deep and rapidly—in order to assess supraglottic movements at rest. Next: Sustained vowel “e,” both a long “eeeee” and several short “e”s—in order to evaluate laryngeal symmetry and motion. Thereafter: Alternate between voluntary making “stridor-like noise” vs. not making “stridor-like noise” during inspiration.

The objective of this part of the protocol is to examine the laryngeal function at rest and to provide the patient with some basic knowledge on laryngeal anatomy and function in a quiet atmosphere.

The CLE test then starts, and will be performed as described above.

#### After the CLE Test

1) Immediately after finishing the CLE test, the mouthpiece will be removed, but the laryngoscope will be kept in place. The patient will then be asked to turn to face the monitor showing the laryngoscopy recording. While still having ongoing symptoms, important structures and landmarks will be pointed out, and the paradoxical movements will be thoroughly described to the patient.
2) Patients in need of guidance to make symptoms abate and patients who panic will carefully be guided through the techniques described below under item 4, in order to obtain control of their laryngeal obstruction.
3) When symptoms have stopped and the patient is able to receive information, there will be given a series of standardized teaching lessons. These lessons will be provided to the patients while the laryngoscope is left in place, in order to allow the patients to observe their larynx on the monitor while performing the exercises listed below.
  a. Reproduction of symptoms: The patients will be asked to repeat what he/she did before the CLE test started; i.e., alternate between intentionally making “stridor-like noise” vs. making no “stridor-like noise” during inspiration while watching the laryngeal movements on the monitor. The goal of this session is to make the patients understand what takes place in their larynx when they make “stridor-like noise” during inspiration. The session will go on for as long as it takes to grasp this information.
  b. Posture: The patients will be explained and shown that keeping the head high and lifted, and the shoulders low and retracted, are the preferred posture to ensure optimal and unobstructed breathing.
  c. Breathing muscles:
    i. It will be explained and shown how the patients should relax their shoulder girdle and perform diaphragmatic breathing rather than clavicular breathing.
    ii. The patients will be asked to breathe by expanding the thorax and the rib cage and to inhale quietly and deeply, attempting to avoid using the muscles around the throat or neck for assistance, while actively being coached to relax the muscles in the shoulder girdle. If necessary, the test leader will put the palm of his/her hand on the patient's chest or on both sides of the lower ribcage and ask the patient to inhale while lifting the arms and the hands.
    iii. Thereafter, the patient will practice the above (item i and ii), producing 5–10 rapid breaths, ensuring that the thorax moves as explained.
    iv. It will be explained and shown that focusing on exhalation rather than on inhalation may also help to redirect focus from clavicular obstructed breathing to diaphragmatic unobstructed breathing.
4) Learning techniques to prevent or stop future EILO episodes.
  a. First of all, the patients will be explained the importance of keeping calm, avoiding increasing the respiratory rate, suppressing the urge to hyperventilate and—importantly—**not panicking**. The patients need to understand that an EILO attack is not deadly or dangerous.
  b. The patients will be explained and shown that forced inspiration might lead to more laryngeal obstruction, and that **focusing on exhalation** might help.
  c. The patients will learn different **breathing techniques** they can use to avoid laryngeal obstruction in situations where they sense that EILO is about to occur:
    i. Sniffing/inhaling through the nose or through closed teeth (Karlsen's maneuver).
    ii. Put the tongue behind the incisors during inhalation.
    iii. Try to yawn and breathe in and out during the yawn.
5) In patients where panicking is part of the problem, the patients will learn that a good posture will help, together with lifting their arms over the head and down again. This maneuver implies lifting the rib cage, thus aiding the inspiration.

The aim is to help the patients to develop a strategy on how to control his/her larynx during exercise, and to be able to continue exercising without experiencing dramatic EILO incidents. The patients will be informed that the best approach is to start practicing while performing low to moderate intensity exercise, and then gradually increase the intensity as they become more confident. It will be emphasized that the new breathing technique they are about to adopt will need to be repeated until it becomes adopted as a part of their automated breathing pattern.

For all three non-invasive treatment groups, there will be a video meeting 1 and 3 weeks after the in-house training session, lasting a maximum of 30 min. The patients will be asked to demonstrate how they perform their breathing practices, and corrected when necessary.

### Inspiratory Muscle Training (IMT)

The inspiratory muscle training (IMT) will build on the information the patients have obtained during the IBA and biofeedback session described above. The IMT will focus on training endurance and coordination of the posterior cricoarytenoid (PCA) muscle, aiming to reduce fatigue of the abducting capacity of the larynx and to enhance coordination and create a sense of laryngeal control. When performing the IMT sessions, it is of utmost importance that a functional diaphragmatic breathing pattern has been established, and that this breathing pattern is maintained throughout all the IMT sessions. Once the patient has demonstrated that he/she is able to perform breathing according to these principles, the IMT session can start according to this flow chart.

#### IMT Teaching Session at the Hospital

1. The maximal inspiratory mouth pressure (PI_max_) will be established, according to the instructions provided by the manufacturer (*POWERbreathe, Southam, UK*) (32). The resistance of the training device is set to 20–60% of maximum PI_max_.
2. A flexible laryngoscope is positioned with its tip in the epipharynx, ensuring a good view of the laryngeal entrance, and secured using a headset.
3. The patient then enters into the IMT teaching session, performed with the laryngoscope in place, with the patient facing the monitor that shows the laryngeal movements while the patient is practicing the IMT.
  a) The training device is positioned correctly in the mouth of the patient.
  b) The patient exhales slowly from TLC to RV, using at least 5 s to reach RV, emphasizing a correct posture, relaxation of potential tensions in the thoracic, shoulder and neck region, and minimal movements of the head and shoulders.
  c) From the RV position, the patient then performs a “determined” inspiration of short duration up to TLC against the resistance of the training device. A correct breathing technique is strongly emphasized; i.e., diaphragmatic breathing with minimal movements of the head and shoulders.
  d) During subsequent repeated training sessions: If TLC appears difficult to reach, it may be a sign of fatigue or an inappropriate breathing pattern.
4. The technique and the pressure settings are adjusted as needed, in order to ensure that the larynx is kept maximally open (abducted) during the inspiration.

#### IMT Performed at Home

1) The patients will perform IMT at home, as they were instructed during the teaching session at the hospital.
2) They will perform 30 loaded breaths twice daily for 5 days per week for 6 weeks with the resistance set to 20–60% of maximum PI_max_.
3) It will be emphasized that it is of utmost importance that the breathing technique explained during the teaching session always must be applied at all subsequent IMT training sessions; i.e., they must ensure diaphragmatic breathing with minimal movements of the head and the shoulders.
4) The IMT device will contain a memory card, thus ensuring that treatment compliance is measured, and that data from all training sessions are stored for later inspection.

There will be a video meeting 1 and 3 weeks after the in-house training session, lasting maximum 30 min. The patients will be asked to demonstrate how they perform their breathing practices, and corrected when necessary.

The patients are advised to use the knowledge they gain about breathing techniques and the experience they gain from the IMT sessions, when they perform exercise at home. The aim is to help the patients to develop a strategy on how to control his/her larynx during exercise, and to be able to continue exercising without experiencing dramatic EILO incidents. They will be informed that the best approach is to start practicing while performing low to moderate intensity exercise, and then gradually increase the intensity as they become more confident. It will be emphasized that the new breathing technique they are about to adopt will need to be repeated until it becomes adopted as a part of their automated breathing pattern.

### Speech Therapy

Speech therapy used to help patients maintain an open larynx when exposed to various triggers of inducible laryngeal obstruction (ILO) has been performed in different ways according to a variety of protocols. In fact, speech therapy has traditionally been considered mainstay therapy for this condition. Nevertheless, the protocols used in most studies are hard to reproduce, as they are often not properly explained in the publications. At our institution, we have gained clinical experience with a local treatment protocol, developed by our speech therapist (TK). This consisting of a 5-set training program split into sessions with guidance by the speech therapist, alternating with individual practice. The lectures can be provided intensively over 3 days, with 1-h sessions 3 times a day the two first days, and one final session the third day, or as 1 h per week with guidance for 5 weeks.

The first session will provide the patients with information about the pathophysiological nature of EILO, as understood from the point of view of a speech therapist, with explanations aided by posters showing the anatomy of the larynx. Moreover, the basic principles of a functional breathing pattern will be explained. Thereafter, the patients will be taken through a standard exercise program for diaphragmatic breathing, on how to obtain and maintain a good posture and on how to release tensions in the larynx. The patient will be trained to actively use the abdominal muscles while exhaling, and to relax the abdominal muscles while releasing air back into the lungs to ensure optimal diaphragmatic activity. It is highlighted that the therapist should have thorough training in the treatment protocol before treating patients. All our speech therapists in the study have this training.

As for the IBA and IMT, the aim of the speech therapy is to help the patients to develop a strategy on how to control his/her larynx during exercise, and to be able to continue exercising without experiencing dramatic EILO incidents. They will be informed that the best approach is to start practicing while performing low to moderate intensity exercise, and then gradually increase the intensity as they become more confident. It will be emphasized that the new breathing technique they are about to adopt will need to be repeated until it becomes adapted as a part of their automated breathing pattern.

For all three non-invasive treatment groups, there will be a video meeting 1 and 3 weeks after the in-house training session, lasting maximum 30 min. The patients will be asked to demonstrate how they perform their breathing practices, and corrected when necessary.

## Phase 3—Surgical Intervention

Surgery is currently available at our institution, irrespective of this treatment protocol, for patients with EILO who continue to exhibit EILO symptoms and still demonstrate supraglottic collapse ≥2 during a CLE test despite non-invasive treatment, and who are highly motivated for further treatment (11). Careful selection of patients adhering to these strict criteria is emphasized in the daily routine clinical work. The inclusion criteria for surgical treatment in the present study (Phase 3) are the same as those applied in the daily routine clinical work. The only exception is that the nature of the non-invasive treatment algorithm is stricter, and surgery is not hampered by availability.

Surgical treatment for severe supraglottic EILO is currently performed according to a procedure originally used to treat severe laryngomalacia (14). We do not know if it is possible to use a less radical or less extensive approach and still obtain sufficient symptom relief. We know from unpublished channels, that some are using less extensive procedures as in-office procedures. However, there are no formal reports from studies using such techniques. A less extensive surgical procedure that efficiently relieves EILO symptoms would clearly be preferable to the current relatively resource-intensive technique.

Thus, the aim of this surgical phase of the protocol is to test effects from two surgical techniques to treat severe supraglottic EILO:

1. Supraglottoplasty—full procedure under general anesthesia.
2. Supraglottoplasty—minimally invasive procedure under general anesthesia.
3. A control group receiving no further treatment, against which both methods will be compared to assess effect.

### Supraglottoplasty—Full Procedure

**Figure 4:**
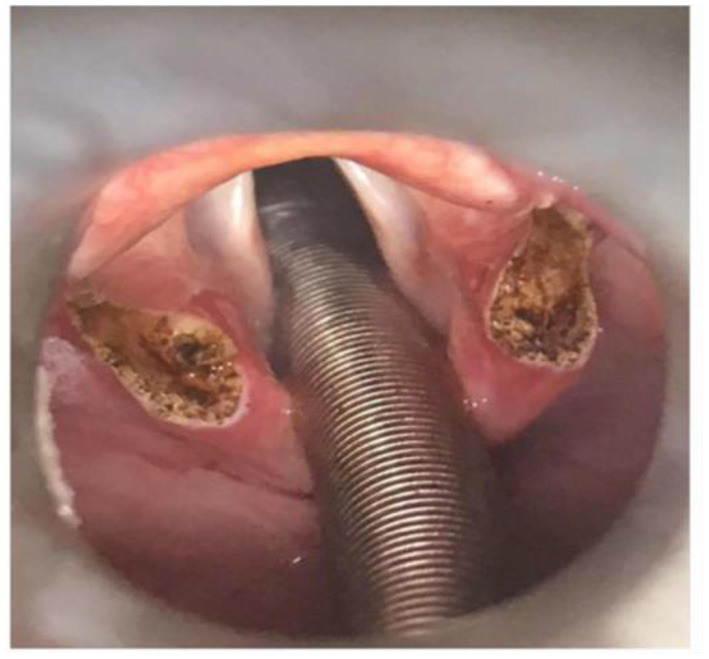
Supraglottoplasty, full procedure, illustrating removal of the cuneiform tubercles on both sides.

The surgeon informs each patient about the objectives of the procedure and the irreversible change of the laryngeal anatomy caused by this operation. The patient should understand the balance between the prospects of improvement and the risk factors involved; i.e., post-operative aspiration, scarring, infection, and bleeding. The supraglottoplasty is performed under general anesthesia by suspension laryngoscopy with use of CO_2_ laser and microlaryngeal instruments. The patient is intubated with an armored laser-tube which is positioned in the posterior midline to protect this area from laser injury. The laryngoscope (Benjamin-Lindholm from Karl Storz) is positioned into the vallecula and the operation field is visualized through a surgical microscope. The CO_2_-laser (Digital Acublade Lumenis) is attached to the microscope through a micromanipulator, laser energy adjusted to 2–4 W and focused with micro spot during the procedure. Releasing incisions are made anteriorly in both aryepiglottic fold. The depth of the incisions are limited to the cranial border of the ventricular fold (Figure 4).

The cranial ends of cuneiform tubercle including their mucosa are removed in a circular pattern before the two incisions are adjoined, thus creating a drop-shaped excision.

Laser surgery dorsal to the corniculate tuberculum is avoided, as scaring in this area could reduce the motility of the posterior part of the aryepiglottic fold. Posterior commissure and the piriform sinus are for this reason protected with wet tissue cloths during the procedure. In case of perioperative oedema of the laryngeal mucosa, corticosteroids are administrated to prevent laryngeal oedema post-operatively. No antibiotic prophylaxis is administered.

### Supraglottoplasty—Minimally Invasive Procedure

The minimally invasive procedure has potential for improving the supraglottic laryngeal obstruction during exercise by “stiffening” the rims of the aryepiglottic fold, thereby preventing inward collapse during high volume ventilation. The method will affect *neither* the anatomical shape/configuration/gross anatomy of the larynx, nor the dimension of the laryngeal inlet at rest. Moreover, the method will not elongate the aryepiglottic fold, and the cuneiform tubercle will not be excised.

The surgeon informs each patient about the objectives of the procedure, as described under the heading “Supraglottoplasty—full procedure” above. The patients are intubated with an armored laser-tube, which is positioned in the posterior midline to protect this area from laser injury. The laryngoscope (Benjamin-Lindholm from Karl Storz) is positioned into the vallecula and the surgical field is visualized through an operation-microscope. The CO_2_-laser (Digital Acublade Lumenis) is attached to the microscope through a micromanipulator, laser energy adjusted to 2–4 W and focused with micro-spot during the procedure. Four punctures are made along the lateral borders of both aryepiglottic fold, thus creating a row of small punctures parallel to the rim of the aryepiglottic fold. The punctions should not be deeper than the incision in the “full procedure” (above); i.e., <5 mm, and care must be taken to avoid thermal damage (e.g., the nervus recurrent posteriorly). It is recommended to protect the posterior commissure and the piriform sinus with wet tissue cloths. No antibiotic prophylaxis is administered. The method is minimally invasive; i.e., if successful, it will in the future be possible to perform this method under local anesthesia.

#### Follow-Up

Patients may be discharged the same day in case of an uneventful procedure. Analgesics may be advised for 3–4 days postoperatively. The patients are advised not to perform hard exercise for the next 10 days. All cases will be scheduled for a follow-up CLE-test with pressure measurement 2–4 months after surgery; however, for participants belonging to the ≪no-surgery control group≫ this will have to take place 2–4 months after surgery would have been performed had they belonged to group E or F (please see Figure 2, Phase 3). Decisions regarding further treatment will rest on the findings from this follow-up, with advice provided individually and in close cooperation with the involved patients. The principles of “shared decision making” will be applied (33).

## Statistical Methods

### Design and Randomization

The main part of the study has a 2 × 2 factorial design with the factors IMT (no/yes) and speech therapy (no/yes). The control arm consists of the patients receiving neither IMT nor speech therapy, only standardized information and breathing advice (IBA). The patients will be randomized in equal numbers to the four arms, using block randomization and the statistical software R. A randomization list will be prepared before the first patient is included. If there is any loss to follow-up, additional patients will be recruited and consecutively assigned to the arm(s) with loss to follow-up. The main analysis will be done on the patients with complete follow-up, but the number of patients lost to follow-up will be reported.

For the surgical sub-study, equal number of patients will be allocated to the three arms, and the randomization will be done using manually shuffled sealed envelopes.

### Statistical Model

The primary outcome is CLE score obtained close to peak exercise, measured on an ordinal 0–6 scale. For the main analysis, a proportional odds (cumulative logit) model will be fitted to the data. The explanatory variables will be the two treatment factors, their interaction and the baseline CLE score (as a continuous/linear variable). If the interaction is clearly insignificant, *P* ≥ 0.20, it will be removed from the model, and the results will be reported for the simplified model. The results will be presented as odds ratios (with the control arm as the reference group) with confidence intervals (CIs). Mean scores for the four arms will also be reported, with CIs calculated using the percentile bootstrap. If the baseline score is statistically significant (*P* ≤ 0.05), estimated model-based probabilities of obtaining the various CLE scores based on baseline CLE score and treatment arm will be reported.

A similar proportional odds model will be fitted for the surgical part of the study, the only difference being three instead of four treatment arms. With the relative small number of patients, there is a risk of non-convergence of the ordinal model (mainly if all patients in an arm end up with the same score). In this case, we will instead use a chi-squared omnibus test, followed by pairwise chi-squared tests. The *P*-values from these pairwise tests will be adjusted for multiple comparisons using Fisher's procedure (34).

There is uncertainty in how the proposed ordinal models will fit the data. We may need to do some adjustments to the model to improve model fit (e.g., include the baseline scores as a quadratic instead of a linear term), or possibly use other statistical models. If no statistical model can be found that adequately fits the data, we will simply report the score distribution in the four/three arms, along with mean scores and corresponding confidence intervals.

### Power Analysis

For practical reasons, we perform the power analysis for a simpler model, where the baseline score is not included. The distribution of CLE scores 2–6 for patients referred to our clinic and meeting the inclusion criteria has been searched in our EILO registry, and found to be 23, 45, 25, 5, and 2%, respectively. We expect that the control group will have a similar distribution, and use this as the basis for the power analysis. We postulate that one of the factors will be effective, with an odds ratio (OR) of 0.3, the other factor will have zero effect (OR = 0) and that there is no interaction. This corresponds to a reduction in mean CLE score of 0.53 for the effective treatment, from 3.18 to 2.65, and is both clinically relevant and in line with effect sizes observed in previous studies (10). The distribution of CLE scores 2–6 will then be 50, 38, 10, 2, and 1%, respectively. (We expect that in practice, some of the patients will have scores below 2, so 2 on this scale should be interpreted as ≤2.) With 35 patients in each arm, computer simulations show that this will give a power of 90% for an overall test of difference between the four arms (even when not using the rule about removing insignificant interactions). An alternative model, where the two treatments have the same effect (OR = 0.3) but there is no additional effect of having both treatments (i.e., a negative interaction) gives a power of 80%. In the actual study, we expect that the baseline score will be a good predictor of the outcome, which will further increase the power.

For the surgical part of the study, the power analysis is similar, with the “no surgery” arm having the same distribution of CLE scores as before (mean score 3.18). However, the effect of the two procedures is postulated as being much larger, OR = 0.05, which corresponds to a mean score in both surgery arms of 2.17. This difference in mean scores of 1.01 is in line with effect sizes from a previous study (11). Including 14 patients in each of the three groups will give a power of ~98% for an overall test (before any adjustment for the baseline scores), and 89% for pairwise comparisons (adjusted for multiple comparisons). The back-up chi-squared test will have a power of 90%. By clinical experience, surgical treatment is provided to ~14% of our EILO population, thus 42 patients entering Phase 3 is a reasonable estimate.

## Ethics

Adverse events will be addressed and recorded throughout the complete study according to the principles for good clinical practice (35).

The CLE test is used routinely at the EILO-clinic at Haukeland University Hospital, which is a nationwide Norwegian reference center for EILO work-up. The test has proven tolerable and safe and has been performed by experienced laboratory personnel on more than 3,000 patients. The pressure sensors, required to measure the translaryngeal pressure drop at peak exercise, represents an add-on to the standard CLE test and increase the diameter of the laryngoscope by 2 mm, therefore slightly increasing the discomfort. The translaryngeal pressure measurement also requires a need for anesthetizing the laryngeal area, which some might find uncomfortable. We have so far performed ~50 CLE tests with pressure transducers, with no adverse events.

The major disadvantages from participating in the study are the extra time required to complete questionnaires and assessment forms, and to perform extra CLE tests that involve some discomfort. It is important in this context that the patients who are invited to take part in this study, all have completed a CLE test before inclusion. They will thus all know by experience what this test requires.

The benefits from participating in the study are primarily that the patients are put through a systematized and structured approach to evaluation and treatment with regular and frequent assessments during the interventional periods, performed at scheduled time points. The routine follow-up provided by the hospital system cannot offer similarly close contact with the patients. It is our clinical impression that close follow-up over time contributes to a better understanding and internalization of the principles of rational breathing techniques. Thus, it is not unreasonable to assume that participation in this project will put our patients in a better position as regards successfully handling their EILO.

## Summary

Given the high prevalence of EILO, the rather limited understanding of the role played by the larynx during exercise and the lack of evidence-based treatment approaches are a cause of concern. We here present our protocol for randomized treatment for EILO. This study will provide information listed as a priority in the Task Force statement issued by three distinguished clinical societies, the ERS, ELS, and ACCP. Knowledge gained from this study is highly requested by clinicians and researchers working in this field of respiratory medicine, and the study protocol meets the requirements listed by the Task Force. Thus, this study represents a first attempt to generate treatment algorithms for EILO based on scientifically robust evidence.

## BergenILO-Group

From Bergen ILO-group, these researchers where particularly involved: Haakon Kvidaland, Department of Pediatric and Adolescent Medicine, Haukeland University Hospital, Bergen, Norway; Petrine Solli, Department of Pediatric and Adolescent Medicine, Haukeland University Hospital, Bergen, Norway; Petter Carlsen, Department of Pediatric and Adolescent Medicine, Haukeland University Hospital, Bergen, Norway; Praveen Muralitharan, Department of Pediatric and Adolescent Medicine, Haukeland University Hospital, Bergen, Norway; Mette Engan, Department of Pediatric and Adolescent Medicine, Haukeland University Hospital, Bergen, Norway; Lorentz Sandvik, Department of Otolaryngology and Head and Neck Surgery, Haukeland University Hospital, Bergen, Norway; Ole C. O. Gamlemshaug, Department of Otolaryngology and Head and Neck Surgery, Haukeland University Hospital, Bergen, Norway.

## Author Contributions

HC, OR, J-HH, MV, and TH contributed to conception and design of the study. KH created the statistical design of the study. HC and TH wrote the first draft of the manuscript. TK, ZF-K, AS, TA, MH, SH, and KH wrote sections of the manuscript. All authors contributed to manuscript revision, read, and approved the submitted version.

## Funding

This study has received a major funding for strategic investment from the Western Norway Regional Health Authority. In addition, we have professors, associated professors, Ph.D. candidates, Master's degree candidates, and Postdoc candidates receiving funding from the University of Bergen, the Western Norway University of Applied Science, Haukeland University Hospital, and the Western Norway Regional Health Authority. The Western Norway Regional Health Authority had no role in the design and conduct of the study.

## Conflict of Interest

The authors declare that the research was conducted in the absence of any commercial or financial relationships that could be construed as a potential conflict of interest. The reviewer CM declared a shared Committee with one the author J-HH at time of review.

## Publisher's Note

All claims expressed in this article are solely those of the authors and do not necessarily represent those of their affiliated organizations, or those of the publisher, the editors and the reviewers. Any product that may be evaluated in this article, or claim that may be made by its manufacturer, is not guaranteed or endorsed by the publisher.
